# Efeitos da Acupuntura na Hipertensão Arterial Sistêmica e na Disfunção Endotelial: Um Ensaio Clínico Randomizado AHEaD

**DOI:** 10.36660/abc.20260462

**Published:** 2026-08-03

**Authors:** Amanda Rodrigues Bitencourt, Ana Luiza Lima Sousa, Paulo Cesar B. Veiga Jardim, Alexandre Massao Yoshizumi, Mikaelle Costa Correia, Priscila Valverde de Oliveira Vitorino, Sandro Rogério Rodrigues Batista, Weimar Kunz Sebba Barroso

**Affiliations:** 1 Universidade Federal de Goiás Unidade de Hipertensão Arterial Goiânia GO Brasil Universidade Federal de Goiás – Unidade de Hipertensão Arterial, Goiânia, GO – Brasil; 2 Hospital Israelita Albert Einstein São Paulo SP Brasil Hospital Israelita Albert Einstein, São Paulo, SP – Brasil; 3 Pontifícia Universidade Católica de Goiás Escola de Ciências Sociais e da Saúde Goiânia GO Brasil Pontifícia Universidade Católica de Goiás – Escola de Ciências Sociais e da Saúde, Goiânia, GO – Brasil

**Keywords:** Hipertensão, Função Endotelial, Acupuntura

## Abstract

**Fundamento:**

A hiperatividade simpática, o estresse oxidativo e a vasodilatação mediada por óxido nítrico (NO) prejudicada são mecanismos fundamentais envolvidos na patogênese da hipertensão arterial sistêmica (HAS). A acupuntura pode reduzir a pressão arterial por meio da modulação da atividade do sistema nervoso autônomo, do aumento da biodisponibilidade de NO, da atenuação das respostas simpatoexcitatórias e da promoção da vasodilatação.

**Objetivo:**

Avaliar se a acupuntura reduz a pressão arterial sistólica periférica (PASp), a pressão arterial diastólica periférica (PADp), a pressão arterial sistólica central (PASc), a pressão arterial diastólica central (PADc) e a velocidade da onda de pulso (VOP), além de melhorar a dilatação mediada por fluxo (DMF).

**Métodos:**

Este ensaio clínico randomizado prospectivo, unicêntrico, placebo-controlado e simples-cego incluiu adultos com pré-hipertensão ou HAS estágio 1, de baixo risco cardiovascular e que não estavam recebendo terapia anti-hipertensiva. Os participantes foram randomizados para um grupo intervenção submetido à acupuntura manual (n = 14) ou para um grupo controle submetido à acupuntura simulada não penetrante (n = 16). Ambos os grupos realizaram duas sessões de 30 minutos por semana durante 12 semanas. O desfecho primário foi a diferença entre os grupos na PASp, medida no início do estudo (V1), após 12 semanas de tratamento (V2) e na visita de final de 30 dias (V3). O nível de significância estatística foi definido como p < 0,05.

**Resultados:**

As características basais foram comparáveis entre os grupos. Em V2, a acupuntura reduziu a PASp em 13,2 mmHg (p = 0,003) e aumentou a DMF em 2,7% (p = 0,016). No grupo controle, a PASp reduziu-se em 4,1 mmHg (p = 0,035), enquanto a DMF aumentou em 1,6% (p = 0,029). Em comparação com o grupo controle, o grupo intervenção apresentou melhorias significativamente maiores na PASp (−9,1 mmHg, p = 0,006) e na DMF (+3,0%, p = 0,037) em V2. Essas diferenças entre os grupos permaneceram significativas em V3 (PASp: −8,4 mmHg, p = 0,038; DMF: +2,9%, p = 0,028).

**Conclusão:**

A acupuntura melhorou significativamente a função endotelial e reduziu a PASp em adultos com pré-hipertensão ou HAS estágio 1 que não estavam recebendo terapia anti-hipertensiva após 12 semanas de tratamento.

## Graphical abstract

**Figura Central:**
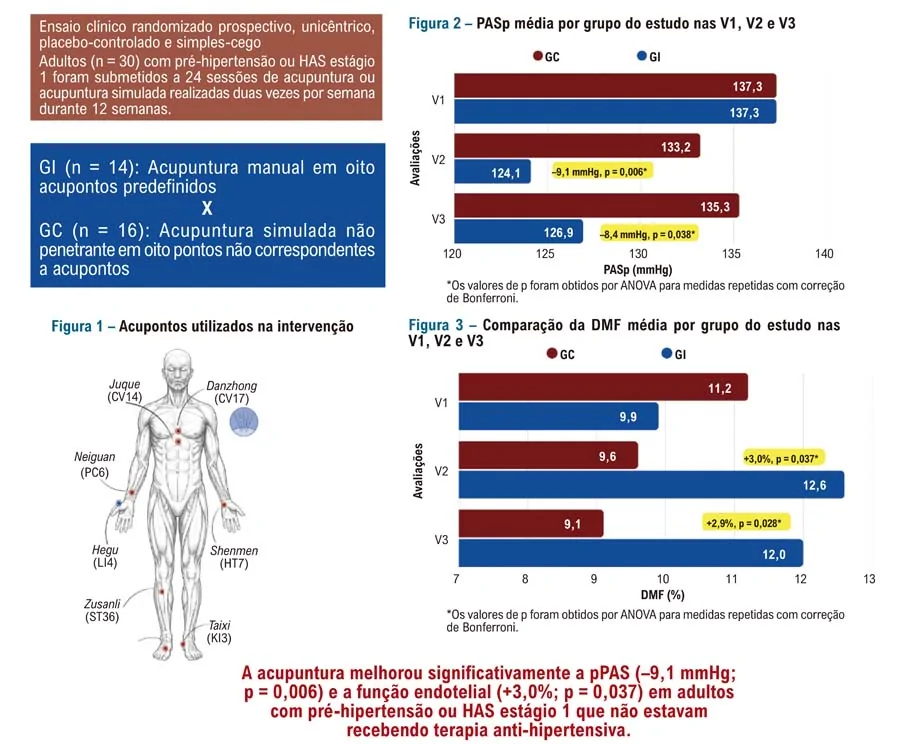
Efeitos da Acupuntura na Hipertensão Arterial Sistêmica e na Disfunção Endotelial: Um Ensaio Clínico Randomizado AHEaD

ANOVA: análise de variância; DMF: dilatação mediada por fluxo; GC: grupo controle; GI: grupo intervenção; HAS: hipertensão arterial sistêmica; PASp: pressão arterial sistólica periférica; V1: visita basal; V2: visita de seguimento; V3: visita final de 30 dias.

## Introdução

A hipertensão arterial sistêmica (HAS) é uma condição crônica multifatorial e de elevada prevalência mundial, sendo um dos principais fatores de risco modificáveis para doenças cardiovasculares (DCV).^1^ Sua prevalência aumenta com a idade e é mais elevada entre indivíduos com mais de 60 anos.^1^ Outros fatores de risco incluem obesidade, sobrepeso, inatividade física, consumo excessivo de sódio e álcool, apneia obstrutiva do sono e poluição do ar.^1^

A fisiopatologia da HAS envolve interações complexas entre fatores genéticos e ambientais que promovem hiperatividade simpática e ativação do sistema renina-angiotensina-aldosterona (SRAA), juntamente com a estimulação de regiões do sistema nervoso central (SNC), como o núcleo paraventricular e a medula rostral ventrolateral (MRVL). Esses mecanismos aumentam a liberação de catecolaminas, angiotensina II, aldosterona e espécies reativas de oxigênio (ROS),^2-4^ resultando em aumento da atividade simpática eferente, intensificação da inflamação e redução da biodisponibilidade de óxido nítrico (NO).^4^ Como consequência, a contratilidade vascular é alterada, ocorre disfunção endotelial e a pressão arterial se eleva.

O aumento do tônus da musculatura lisa vascular, a proliferação de células musculares lisas vasculares na túnica média e o aumento da permeabilidade endotelial, características marcantes da redução da biodisponibilidade de NO, contribuem para o remodelamento vascular subjacente às complicações cardíacas, cerebrais e renais associadas à HAS. A disfunção endotelial está presente em indivíduos com HAS mesmo antes de se tornarem aparentes evidências estruturais de doença aterosclerótica, tornando a avaliação da função endotelial uma ferramenta clínica valiosa.^1,4^

Apesar da ampla disponibilidade de medicamentos anti-hipertensivos eficazes, as taxas de controle da HAS permanecem insatisfatórias em todo o mundo, em grande parte devido à baixa adesão ao tratamento.^1^ Assim, intervenções não farmacológicas são recomendadas para todos os indivíduos com HAS.^1,5^

A acupuntura pode proporcionar efeitos benéficos em pacientes com HAS.^6-10^ O estímulo nociceptivo desencadeado pela inserção das agulhas alcança os neurônios simpáticos pré-ganglionares na MRVL, inibindo assim os reflexos simpatoexcitatórios viscerais e reduzindo o espasmo da musculatura lisa vascular.^6,11-14^ A acupuntura também suprime a ativação simpática central mediada pelo SRAA,^13^ reduz a secreção de renina, a atividade dos receptores de angiotensina II,^12,13^ e a produção de ROS, além de ativar a NO sintase endotelial. Esses mecanismos aumentam a biodisponibilidade de NO, promovem vasodilatação e melhoram a disfunção endotelial.^6,14,15^ Com base nesses mecanismos,^6,11-14^ levantamos a hipótese de que a acupuntura poderia reduzir a pressão arterial e melhorar a função endotelial. No entanto, as evidências disponíveis permanecem inconsistentes quanto ao seu papel como tratamento não farmacológico da HAS^10,16^ e quanto aos seus efeitos sobre outros preditores cardiovasculares, incluindo rigidez arterial e disfunção endotelial.

Assim, o presente ensaio clínico randomizado (ECR) teve como objetivo avaliar os efeitos da acupuntura, em comparação com um grupo controle, sobre a pressão arterial sistólica periférica (PASp), a pressão arterial diastólica periférica (PADp), a pressão arterial sistólica central (PASc), a pressão arterial diastólica central (PADc), a velocidade da onda de pulso (VOP) e a dilatação mediada por fluxo (DMF) em pacientes com pré-hipertensão ou HAS estágio 1 após 24 sessões de tratamento.

## Métodos

### Delineamento do estudo e cenário

Este ECR prospectivo, unicêntrico, placebo-controlado e simples-cego foi conduzido no centro de pesquisa da Unidade de Hipertensão Arterial da Faculdade de Medicina da Universidade Federal de Goiás (UFG), Brasil.

O estudo foi registrado no Registro Brasileiro de Ensaios Clínicos (sob o código de registro RBR-9qnxb9z), conduzido em conformidade com as diretrizes Revised STandards for Reporting Interventions in Clinical Trials of Acupuncture e CONsolidated Standards Of Reporting Trials,^17^ e aprovado pelo Comitê de Ética em Pesquisa do Hospital das Clínicas da UFG (parecer nº 6.244.770). Todos os participantes forneceram consentimento informado por escrito antes da inclusão no estudo.

### População do estudo

A população do estudo foi composta por adultos com pré-hipertensão ou HAS estágio 1, de baixo risco cardiovascular e que não estavam recebendo terapia anti-hipertensiva.

Os critérios de exclusão foram HAS estágio 2 ou 3; risco cardiovascular moderado ou alto; uso de medicamentos conhecidos por afetar a pressão arterial; diabetes melito; nefropatia; histórico de doença arterial coronariana e/ou doença cerebrovascular; insuficiência cardíaca; distúrbios da tireoide; qualquer condição que comprometesse a condução neural; discrasias sanguíneas graves; lesões cutâneas ou outras condições que impedissem o acesso aos acupontos; gestação ou lactação; transtornos psiquiátricos não controlados; tratamento farmacológico anti-hipertensivo nos 6 meses anteriores; ou histórico prévio de acupuntura.

### Cálculo do tamanho da amostra

O tamanho da amostra foi calculado para a comparação da média da PASp entre os grupos intervenção e controle, assumindo uma diferença mínima clinicamente relevante de 3 mmHg. Com base em um nível de significância bilateral de 5%, poder estatístico de 90% e uma taxa de perdas prevista de 10%, o tamanho amostral necessário foi estimado em 25 participantes por grupo.^7^

### Recrutamento e randomização dos participantes

Os participantes foram recrutados a partir do banco de dados do centro de pesquisa da Unidade de Hipertensão Arterial entre março de 2024 e fevereiro de 2025. Os indivíduos que atenderam a todos os critérios de inclusão e a nenhum dos critérios de exclusão foram contatados por telefone e convidados a comparecer à visita basal (V1).

Após a inclusão no estudo, todos os participantes foram submetidos às avaliações em V1, na visita de seguimento (V2) e na visita de encerramento do estudo (V3), realizada 30 dias após a conclusão da intervenção.

### Visita basal

Todos os participantes foram avaliados pela mesma cardiologista e medica acupunturiatra certificada, incluindo anamnese e exame físico. O estágio da HAS foi confirmado utilizando o esfigmomanômetro automatizado Omron HBP-1100, de acordo com as Diretrizes Brasileiras de Hipertensão Arterial – 2020.^1^ Foram considerados elegíveis os indivíduos com PASp de 130-159 mmHg e/ou PADp de 85-99 mmHg.

A pressão arterial periférica foi aferida após 5 minutos de repouso, com os participantes sentados, pernas descruzadas e braços apoiados. A pressão arterial foi medida em ambos os braços utilizando o esfigmomanômetro Omron HBP-1100, e o braço com o maior valor foi utilizado para todas as medidas subsequentes, que incluem VOP, PASp, PADp, PASc, PADc e DMF. Três medidas consecutivas foram obtidas com intervalo de 1 minuto entre elas, sendo utilizada a média das duas últimas para classificar o estágio da HAS. Os valores finais foram registrados em um formulário padronizado de relato de caso.

Após a confirmação da elegibilidade e a assinatura do termo de consentimento informado por escrito, os participantes foram randomizados para o grupo intervenção (acupuntura manual) ou para o grupo controle (acupuntura simulada não penetrante), utilizando uma ferramenta de randomização baseada na *web*.^18^ A alocação foi realizada de forma consecutiva e sequencial no momento da inclusão por um pesquisador que não participou do recrutamento, da avaliação ou da aplicação da intervenção.

A função endotelial, avaliada por meio da DMF, juntamente com a PASc, PADc e VOP, foi examinada em todos os participantes. As avaliações pela mesma cardiologista e medica acupunturiatra certificada, em uma sala silenciosa e com temperatura controlada, entre 7h00 e 11h00, de acordo com as diretrizes vigentes.^19,20^

A pressão arterial central (avaliada por meio da PASc e da PADc) e a rigidez arterial (avaliada por meio da VOP) foram mensuradas utilizando o dispositivo Mobil-O-Graph^®^ (IEM GmbH, Stolberg, Alemanha).^19^

A função endotelial foi avaliada utilizando o sistema de ultrassonografia de alta resolução UNEX EF38G, considerado o padrão de referência para a mensuração da DMF. O equipamento incorpora um braço robótico que realiza o escaneamento automático da artéria braquial com elevada precisão e reprodutibilidade. Um manguito pneumático posicionado distalmente à fossa antecubital foi insuflado até 50 mmHg acima da PASp do participante durante 5 minutos. O diâmetro longitudinal da artéria braquial foi registrado continuamente por ultrassonografia durante 60 segundos imediatamente antes da insuflação do manguito e durante 2 minutos após sua desinsuflação.^20^

### Visitas de seguimento e de encerramento do estudo

A V2 foi realizada após os participantes completarem as 24 sessões de intervenção. A V3 foi realizada 30 dias após a V2. Em ambas as visitas, foram realizadas avaliação clínica e mensurações da PASp, PADp, PASc, PADc, VOP e função endotelial por meio da DMF, de acordo com o mesmo protocolo utilizado em V1, pela mesma pesquisadora.

Durante a V3, os participantes foram convidados a identificar o tipo de agulha que acreditavam ter sido utilizada durante a intervenção, escolhendo entre uma agulha penetrante na pele (acupuntura manual) e uma agulha não penetrante (agulha placebo de Streitberger).^21,22^

### Intervenção

#### Grupos intervenção e controle

Os participantes alocados no grupo intervenção receberam acupuntura manual utilizando oito agulhas estéreis de aço inoxidável (0,25 × 30 mm), aplicadas de acordo com a teoria dos meridianos da medicina tradicional chinesa (MTC),^23^ as recomendações do Colégio Médico Brasileiro de Acupuntura e as diretrizes da Organização Mundial da Saúde.^24^ Todos os procedimentos foram realizados por medica acupunturiatra com 5 anos de experiência clínica. Foram utilizados oito acupontos: PC6, LI4, HT7, CV14, CV17, ST36, LR3 e KI3 (Figura 1).^25^

**Figura 1:**
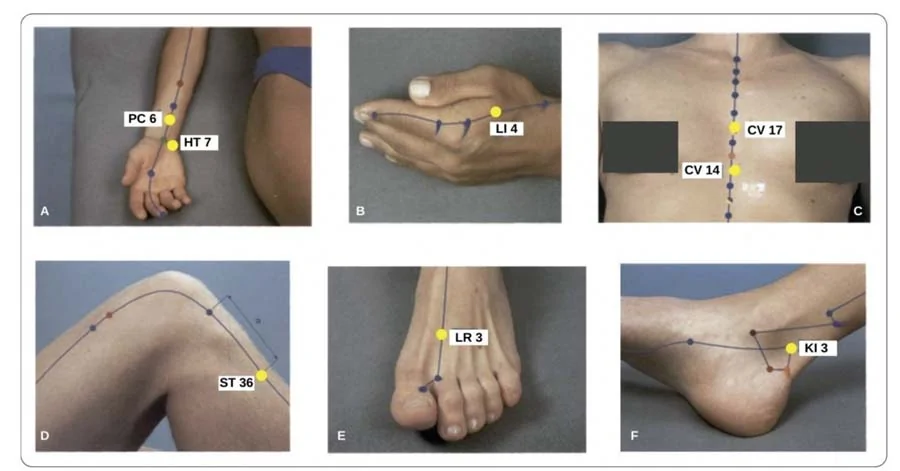
– Acupontos utilizados na intervenção. A) PC6 (Neiguan) e HT7 (Shenmen); B) LI4 (Hegu); C) CV14 (Juque) e CV17 (Danzhong); D) ST36 (Zusanli); E) LR3 (Taichong); e F) KI3 (Taixi). Adaptado de Atlas Gráfico de Acupuntura (ISBN: 9783833156878).

As agulhas foram inseridas ao longo da direção do meridiano correspondente, em um ângulo de 30° a 60°, sem estimulação manual, permanecendo no local por 30 minutos em cada sessão. Os participantes não foram questionados quanto à sensação provocada pela agulha (*De qi*). A profundidade de inserção das agulhas variou, em geral, de 1,0 a 1,5 cm, dependendo da espessura da pele e das características do tecido subcutâneo. Oito agulhas foram utilizadas em cada sessão de tratamento. Os acupontos localizados nos membros superiores (PC6, LI4 e HT7) e nos membros inferiores (ST36, LR3 e KI3) foram aplicados unilateralmente, no lado esquerdo ou direito, sendo o lado selecionado aleatoriamente para cada sessão.

Os acupontos foram escolhidos com base em sua relevância terapêutica para a HAS segundo a teoria da MTC,^23^ em seus efeitos neurofisiológicos^26-30^ e em suas frequencias de uso em ECRSs anteriores^7,9,15^ (Tabela S1 no Arquivo Suplementar 1).

Os participantes alocados no grupo controle receberam oito agulhas de acupuntura simulada não penetrantes (agulhas placebo de Streitberger),^21,22^ aplicadas pela mesma medica acupunturiatra em oito pontos não correspondentes a acupontos, localizados no tronco e nos membros. Após a aplicação, as agulhas permaneceram posicionadas por 30 minutos, sem estimulação manual. Essas agulhas placebo entram em contato com a epiderme, mas não penetram a pele nem estimulam os tecidos somáticos mais profundos, uma vez que a haste da agulha retrai para o interior do cabo durante a aplicação.

Todos os participantes realizaram sessões de tratamento com duração de 30 minutos duas vezes por semana durante 12 semanas consecutivas (24 sessões no total), com intervalo máximo de 3 dias entre as sessões. Durante cada sessão, os participantes utilizaram uma venda ocular individual para impedir a identificação visual da alocação do tratamento e assinaram uma lista de presença. A participação em mais de 80% das sessões programadas foi considerada satisfatória para inclusão na análise dos dados.

## Análise estatística

As análises estatísticas foram realizadas utilizando o IBM SPSS Statistics for Windows, versão 26 (IBM Corp., Armonk, NY, EUA). As variáveis categóricas são apresentadas como frequências e porcentagens, e as associações foram avaliadas pelo teste do qui-quadrado ou pelo teste exato de Fisher quando as frequências esperadas nas células foram ≤ 6. As variáveis contínuas são apresentadas como médias e desvios padrão. O teste de Shapiro-Wilk foi utilizado para avaliar a normalidade da distribuição dos dados, e o teste *t* de Student para amostras independentes foi utilizado para comparar os valores basais entre os grupos intervenção e controle.

Todas as análises foram conduzidas de acordo com o princípio da intenção de tratar (ITT), incorporando os dados das avaliações basal e de seguimento.

Foi realizada análise de variância (ANOVA) unidirecional para medidas repetidas para avaliar o efeito da intervenção sobre a PASp, desfecho primário, nos três momentos de avaliação. A suposição de esfericidade foi avaliada por meio do teste de Mauchly. Quando essa suposição foi violada (p < 0,05), aplicou-se a correção de Greenhouse-Geisser. Quando foi identificado um efeito principal significativo, realizaram-se comparações *post hoc* com ajuste de Bonferroni para determinar os momentos em que ocorreram diferenças significativas.

A confiabilidade interavaliadores das medidas de DMF foi avaliada por meio do coeficiente de correlação intraclasse (CCI), com base em um modelo de efeitos mistos bidirecional com concordância absoluta.^31^

Todos os testes estatísticos foram bicaudais, e o nível de significância estatística foi definido como p < 0,05. As estimativas são apresentadas com intervalos de confiança de 95% (IC95%). Um dos autores teve acesso integral a todos os dados do estudo e assume a responsabilidade pela integridade dos dados e pela exatidão da análise estatística.

## Considerações éticas

Os detalhes são apresentados no Arquivo Suplementar 2.

## Resultados

Dos 133 indivíduos avaliados para elegibilidade, 30 atenderam a todos os critérios de elegibilidade e foram incluídos no estudo, sendo 16 participantes alocados para o grupo controle e 14 para o grupo intervenção. A idade média foi de 42,0 ± 11,4 anos. As características basais, incluindo sexo, índice de massa corporal (IMC), níveis de atividade física (AF), pressão arterial periférica e central, VOP e DMF, foram homogêneas entre os grupos (Tabela 1).

**Tabela 1:** – Características basais dos participantes do estudo em V1

Característica	Controle (n = 16)	Intervenção (n = 14)	Valor de p
**Sexo**			0,464*
Feminino	9 (56,3)	6 (42,9)	
Masculino	7 (43,8)	8 (57,1)	
**AF**			1,000*
Sim	8 (50,0)	7 (50,0)	
Não	8 (50,0)	7 (50,0)	
**IMC**	30,1 ± 4,7	30,1 ± 5,2	0,984^†^
**PASp, mmHg**	137,3 ± 6,5	137,3 ± 8,6	0,992^†^
**PADp, mmHg**	86,8 ± 4,4	86,4 ± 6,0	0,812^†^
**PASc, mmHg**	129,0 ± 12,2	130,5 ± 14,0	0,756^†^
**PADc, mmHg**	90,1 ± 5,9	92,3 ± 8,5	0,408^†^
**VOP, m/s**	6,7 ± 1,2	6,7 ± 1,1	0,940^†^
**DMF, %**	11,2 ± 3,4	9,9 ± 4,6	0,417^†^

V1: visita basal; AF: atividade física; IMC: índice de massa corporal; PASp: pressão arterial sistólica periférica; PADp: pressão arterial diastólica periférica; PASc: pressão arterial sistólica central; PADc: pressão arterial diastólica central; VOP: velocidade da onda de pulso; DMF: dilatação mediada por fluxo; DP: desvio padrão. *Valor de p obtido pelo teste do qui-quadrado; ^†^Valor de p obtido pelo teste t de Student para amostras independentes. Os dados são apresentados como média ± DP ou n (%), conforme apropriado.

Todos os participantes completaram as avaliações em V1, V2 e V3. As taxas de comparecimento às sessões foram de 99,7% no grupo intervenção e de 94,7% no grupo controle. Apenas um participante apresentou taxa de comparecimento (54%) inferior ao limiar preestabelecido (> 80%), mas foi mantido na análise de acordo com o princípio da ITT.

Todos os participantes do grupo intervenção acreditaram ter recebido agulhas penetrantes na pele. No grupo controle, apenas dois participantes identificaram corretamente que haviam recebido agulhas não penetrantes; a exclusão desses participantes não alterou os achados do estudo. Não foram relatados eventos adversos graves. Um participante do grupo intervenção relatou dor leve nos locais de inserção das agulhas.

O IMC e os níveis de AF permaneceram homogêneas entre os grupos em V1, V2 e V3.

A Figura 2 e a Figura 3 apresentam os principais achados. Ambos os grupos apresentaram reduções na PASp após a intervenção de 12 semanas; entretanto, a redução foi significativamente maior no grupo intervenção do que no grupo controle. Além disso, o grupo intervenção manteve essa redução em V3, enquanto nenhum efeito sustentado foi observado no grupo controle. Da mesma forma, a DMF aumentou significativamente no grupo intervenção em comparação com o grupo controle após a intervenção, e essa melhora foi mantida em V3 (Figura Central).

**Figura 2:**
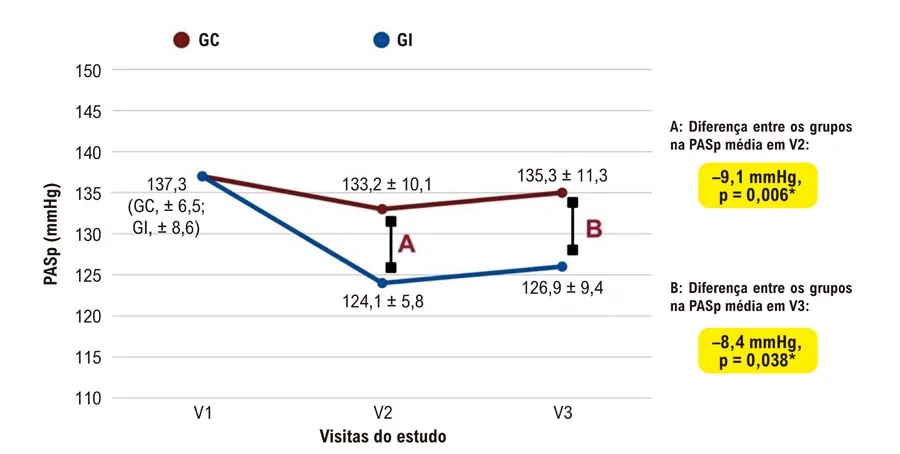
– PASp média por grupo do estudo em V1, V2 e V3. As diferenças entre os grupos na PASp média são apresentadas para V2 (A) e V3 (B). *Os valores de p foram obtidos por ANOVA para medidas repetidas com correção de Bonferroni. ANOVA: análise de variância; GC: grupo controle; GI: grupo intervenção; PASp: pressão arterial sistólica periférica; V1: visita basal; V2: visita de seguimento; V3: visita de final de 30 dias.

**Figura 3:**
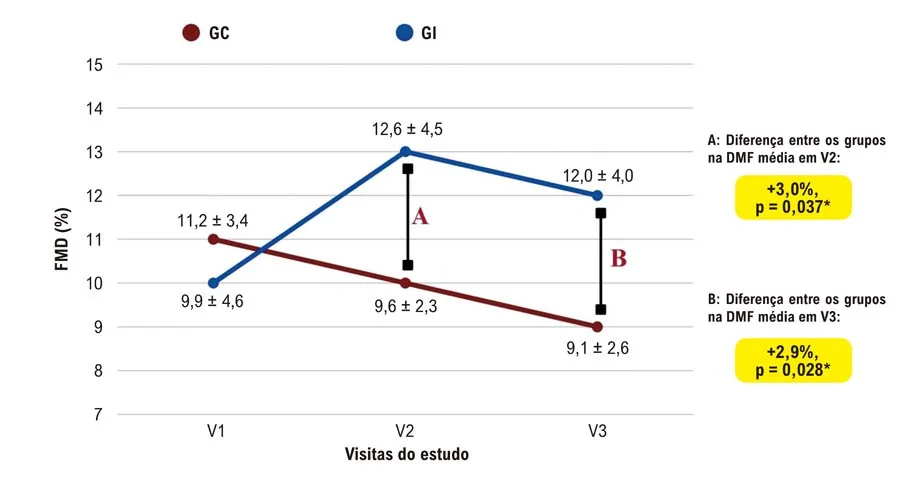
– Comparação da DMF média por grupo do estudo em V1, V2 e V3. As diferenças entre os grupos na DMF média são apresentadas para V2 (A) e V3 (B). *Os valores de p foram obtidos por ANOVA para medidas repetidas com correção de Bonferroni. ANOVA: análise de variância; GC: grupo controle; DMF: dilatação mediada por fluxo; GI: grupo intervenção; V1: visita basal; V2: visita de seguimento; V3: visita de final de 30 dias.

Em V2, os participantes do grupo intervenção apresentaram redução de 13,2 mmHg na PASp e aumento de 2,7% na DMF. Em contrapartida, o grupo controle apresentou redução de 4,1 mmHg na PASp e aumento de 1,6% na DMF. As comparações entre os grupos em V2 demonstraram melhora significativamente maior no grupo intervenção para a PASp (−9,1 mmHg) e a DMF (+3,0%). Em V3, o grupo intervenção manteve melhora significativamente maior na PASp (−8,4 mmHg) e na DMF (+2,9%) em comparação com o grupo controle (Tabela 2).

**Tabela 2:** – Comparação dos valores médios entre e dentro dos grupos do estudo em V1, V2 e V3

Característica	Controle (n = 16)	Valor de p^*^ (dentro do grupo)	Intervenção (n = 14)	Valor de p^*^ (dentro do grupo)	Valor de p^**†**^ (entre os grupos)
**PASp**		0.018			
V1	137,3 ± 6,5		137,3 ± 8,6		0,992
V2	133,2 ± 10,1	0,035^a^	124,1 ± 5,8	0,003^a^	0,006
V3	135,3 ± 11,3	1,000^b^	126,9 ± 9,4	0,017^b^	0,038
**PADp**		0,751			
V1	86,8 ± 4,4		86,4 ± 6,0		0,812
V2	82,2 ± 7,4	0,003^a^	81,6 ± 6,5	0,002^a^	0,823
V3	84,7 ± 9,4	0,760^b^	83,0 ± 5,9	0,147^b^	0,559
**PASc**		0,439			
V1	129,0 ± 12,2		130,5 ± 14,0		0,756
V2	126,2 ± 13,1	0,973^a^	123,0 ± 9,2	0,277^a^	0,453
V3	125,9 ± 10,5	0,793^b^	128,6 ± 14,8	1,000^b^	0,576
**PADc**		0,728			
V1	90,1 ± 5,9		92,3 ± 8,5		0,408
V2	86,2 ± 8,1	0,197^a^	88,7 ± 7,3	0,418^a^	0,390
V3	86,0 ± 8,0	0,097^b^	90,1 ± 9,3	1,000^b^	0,199
**VOP**		0,083			
V1	6,7 ± 1,2		6,7 ± 1,1		0,940
V2	6,7 ± 1,3	1,000^a^	6,4 ± 1,0	0,056^a^	0,502
V3	6,7 ± 1,3	1,000^b^	6,6 ± 1,3	1,000^b^	0,873
**DMF**		< 0,001			
V1	11,2 ± 3,4		9,9 ± 4,6		0,417
V2	9,6 ± 2,3	0,029^a^	12,6 ± 4,5	0,016^a^	0,037
V3	9,1 ± 2,6	0,114^b^	12,0 ± 4,0	0,098^b^	0,028

V1: visita basal; V2: visita de seguimento; V3: visita final de 30 dias; PASp: pressão arterial sistólica periférica; PADp: pressão arterial diastólica periférica; PASc: pressão arterial sistólica central; PADc: pressão arterial diastólica central; VOP: velocidade da onda de pulso; DMF: dilatação mediada por fluxo; ANOVA: análise de variância; DP: desvio padrão. *Valores de p obtidos por ANOVA para medidas repetidas com correção de Bonferroni para as comparações entre V1 e V2 (a) e entre V1 e V3 (b); †Valores de p obtidos por ANOVA para medidas repetidas com correção de Bonferroni para as comparações entre os grupos. Os dados dos grupos intervenção e controle são apresentados como média ± DP.

Foi observada uma interação significativa grupo × tempo para a PASp, indicando que as mudanças ao longo do tempo diferiram entre os grupos intervenção e controle. Também foi observada uma interação significativa grupo × tempo para a DMF (Tabela 2).

A ANOVA para medidas repetidas demonstrou uma redução significativamente maior da PASp ao longo dos três momentos de avaliação no grupo intervenção em comparação com o grupo controle (grupo intervenção: F = 10,964, p = 0,006, η^2^ = 0,458; grupo controle: F = 2,276, p = 0,120, η^2^ = 0,132).

## Discussão

Os achados deste ECR demonstraram melhorias significativas na PASp e na DMF após 12 semanas de intervenção, com esses efeitos persistindo 30 dias após a interrupção do tratamento. Embora o objetivo primário tenha sido avaliar os efeitos da acupuntura sobre a pressão arterial, e não desfechos clínicos, as melhoras na PASp e na DMF podem ter relevância prognóstica, em concordância com estudos anteriores.^4,20,32^ Uma redução de 5 mmHg na PASp tem sido associada a reduções aproximadas de 10% e 8% no risco de eventos cardiovasculares maiores e de doença cardíaca isquêmica, respectivamente.^32^

Flachskampf et al.^9^ relataram reduções significativas na pressão arterial sistólica e diastólica ambulatorial de 24 horas (−6,4 e −3,7 mmHg, respectivamente; p < 0,001), bem como na PASp diurna (−7,3 mmHg; p < 0,001), entre 72 pacientes com HAS estágio 1 ou 2 submetidos a 22 sessões de acupuntura ao longo de 6 semanas em três acupontos bilaterais, em comparação com 68 pacientes que receberam acupuntura simulada com agulhas penetrantes. Embora 75% dos participantes estivessem recebendo terapia anti-hipertensiva estável, uma análise de subgrupo envolvendo 35 participantes que não estavam recebendo tratamento farmacológico (19 no grupo acupuntura) demonstrou reduções sustentadas na pressão arterial média de 24 horas (−6,6 mmHg para a PASp, p = 0,018; e −3,7 mmHg para a PADp, p = 0,027). De forma semelhante, a acupuntura reduziu a PASp no presente estudo. Entretanto, diferentemente de Flachskampf et al.,^9^ não realizamos monitorização ambulatorial da pressão arterial de 24 horas, não observamos efeito significativo sobre a PADp, incluímos apenas participantes que não estavam recebendo terapia anti-hipertensiva e avaliamos um período de intervenção mais prolongado (12 semanas). Além disso, utilizamos agulhas não penetrantes para acupuntura simulada, as quais minimizam os efeitos fisiológicos das agulhas e todos os participantes do grupo intervenção receberam tratamento nos mesmos oito acupontos predefinidos.

De forma semelhante, Liu et al.^7^ relataram redução da PASp média (−8,6 mmHg; p = 0,031) quatro semanas após a conclusão de uma intervenção de acupuntura com duração de 8 semanas em 15 pacientes com pré-hipertensão ou HAS estágio 1, em comparação com controles não tratados. Em concordância com esses achados, observamos uma redução sustentada da PASp média no grupo intervenção 30 dias após a conclusão da intervenção (−8,4 mmHg; p = 0,038). Apesar das diferenças no delineamento do estudo e na avaliação dos desfechos, a redução sustentada da pressão arterial observada após a interrupção do tratamento em ambos os estudos sustenta a hipótese de que a acupuntura possa produzir efeitos anti-hipertensivos persistentes.

A redução média entre os grupos de 9,1 mmHg na PASp observada com a acupuntura pode ser clinicamente relevante, pois é comparável às reduções relatadas em ECRs que avaliaram monoterapia anti-hipertensiva.^33,34^ Uma revisão sistemática de 46 ECRs comparando nove bloqueadores dos receptores da angiotensina com placebo relatou reduções de 6-10 mmHg na PASp.^33^ Da mesma forma, uma metanálise de 60 ECRs encontrou redução média de 9,1 mmHg na PASp com diuréticos tiazídicos em comparação com placebo.^34^

A redução da PASp observada no grupo controle (−4,1 ± 10,1 mmHg; p = 0,035) pode ser atribuída, pelo menos em parte, a um efeito placebo, esperado em praticamente qualquer intervenção terapêutica. A magnitude desse efeito depende de diversos fatores, incluindo a via de administração, o ambiente de tratamento e a relação terapêutica entre o participante e o profissional.^35^ Como ambos os grupos foram tratados em condições idênticas (p.ex., mesmo ambiente clínico, mesmo profissional, mesmo cronograma de tratamento, mesmo número de sessões e mesmo número de locais de estimulação), diferindo apenas quanto ao uso de agulhas penetrantes ou não penetrantes, seria esperado um efeito placebo semelhante em ambos os grupos. Assim, a redução significativamente maior da PASp média no grupo intervenção em comparação com o grupo controle (−9,1 mmHg; p = 0,006), juntamente com a melhora da função endotelial, sugere que os efeitos terapêuticos da acupuntura foram além daqueles atribuíveis ao efeito placebo.

Embora a avaliação da DMF não seja realizada rotineiramente na prática clínica, ela constitui uma ferramenta valiosa para avaliar os efeitos de curto e longo prazo de intervenções farmacológicas e não farmacológicas. A melhora da DMF reflete melhora da função endotelial e está associada à proteção contra doença aterosclerótica e DCV.^36^

Em concordância com os achados de Park et al.,^15^ nossos resultados demonstraram melhora da função endotelial após a acupuntura, potencialmente mediada pelo aumento da ativação da NO sintase endotelial e da produção de NO,^14^ juntamente com a modulação da atividade simpática.^11,12^ Em seu estudo que avaliou os efeitos agudos de quatro sessões de acupuntura em ST36 isoladamente ou combinado com PC6 sobre a DMF em 15 pacientes com HAS em uso de terapia anti-hipertensiva, Park et al.^15^ relataram um aumento significativamente maior da DMF no grupo acupuntura em comparação com o grupo placebo. Entretanto, diferentemente do nosso estudo, foram utilizadas agulhas penetrantes no grupo controle, e a DMF foi avaliada durante um período mais curto após a hiperemia reativa da artéria braquial (60 segundos).

Até onde sabemos, o ensaio AHEaD é o primeiro a demonstrar melhora sustentada da função endotelial após a acupuntura utilizando os mesmos acupontos predefinidos para todos os participantes, um grupo controle com acupuntura simulada não penetrante e medidas de DMF obtidas com um sistema de ultrassonografia de alta resolução equipado com braço robótico automatizado, considerado o padrão de referência para a avaliação da DMF.^20^

A DMF da artéria braquial é um preditor independente de DCV,^4,20^ e a persistência de DMF comprometida, apesar do tratamento otimizado, está associada a maior risco de eventos cardiovasculares futuros.^36^ Portanto, a melhora da DMF observada após a acupuntura pode ter importantes implicações clínicas. Uma metanálise demonstrou que cada aumento de 1% na DMF da artéria braquial está associado a uma redução de 8% a 13% no risco de DCV.^36^

A redução da PASp observada após a acupuntura no presente estudo contrasta com os achados do estudo SHARP.^16^ No SHARP, 12 sessões de acupuntura administradas ao longo de 6-8 semanas em pacientes com HAS estágio 1 ou 2 não reduziram significativamente a PASp em comparação com a acupuntura simulada. Aproximadamente 70% dos participantes daquele estudo haviam recebido previamente terapia anti-hipertensiva, que foi descontinuada antes da randomização, e a intervenção simulada envolveu agulhas penetrantes na pele. Em contraste, nosso estudo utilizou agulhas simuladas não penetrantes, eliminando, assim, o estímulo nociceptivo da agulha nos tecidos profundos. Além disso, nossa intervenção incluiu um maior número de sessões de tratamento, recrutou participantes que não haviam recebido terapia anti-hipertensiva nos 6 meses anteriores e teve duração de 12 semanas, em consonância com as recomendações atuais para o manejo não farmacológico da HAS.^1,5^

Terenteva et al.^37^ relataram melhora da pressão arterial central e dos parâmetros de rigidez arterial após 8 semanas de acupuntura (três sessões por semana), utilizando sete acupontos bilaterais em 23 participantes, apesar da ausência de cegamento dos participantes e de um grupo controle placebo. Em contraste, a acupuntura não melhorou os parâmetros de rigidez arterial no presente estudo. Essa discrepância pode ser explicada por diferenças na frequência do tratamento (duas versus três sessões por semana), no número de acupontos utilizados e pelo tamanho relativamente pequeno da nossa amostra. Na HAS, o enrijecimento arterial resulta de processos moleculares, celulares e mecânicos complexos que comprometem a distensibilidade arterial e aumentam a espessura íntima-média.^19^ A acupuntura pode exercer maior efeito sobre a função endotelial do que sobre o remodelamento da musculatura lisa vascular associado à fibrose e ao enrijecimento arterial, por meio do aumento da biodisponibilidade de NO e da promoção da vasodilatação. Estudos adicionais são necessários para esclarecer os efeitos da acupuntura sobre a rigidez arterial.

O tratamento não farmacológico é recomendado para todos os indivíduos com HAS.^1,5^ A restrição de sódio e álcool, a adesão a um padrão alimentar saudável, a prática regular de AF e intervenções baseadas na espiritualidade demonstraram reduzir a PASp em 5,5-12,0 mmHg.^38-41^ No presente estudo, a acupuntura promoveu reduções na PASp e melhora da DMF de magnitude comparável às relatadas para outras intervenções não farmacológicas, sugerindo que a acupuntura pode representar uma opção adicional de tratamento não farmacológico para a HAS.

Em ECRs, a baixa adesão à intervenção pode comprometer a validade e a interpretação dos achados.^42^ No presente estudo, a adesão foi elevada em ambos os grupos, sem diferenças significativas entre eles. Isso provavelmente reflete o fato de que todas as sessões de tratamento foram conduzidas pela mesma pesquisadora, um clínico experiente especializado no manejo da HAS, que também ofereceu flexibilidade de horários. A elevada taxa de adesão, juntamente com a manutenção de características basais comparáveis ao longo do estudo para variáveis que poderiam influenciar a pressão arterial, sustenta a interpretação de que os efeitos observados foram atribuíveis à intervenção.

A redução entre os grupos na PASp média observada no grupo intervenção (−9,1 mmHg; p = 0,006) foi superior à relatada em uma metanálise anterior.^10^ Esse achado pode ser explicado pela elevada taxa de adesão, pelo uso de agulhas simuladas não penetrantes no grupo controle, pelo protocolo padronizado de acupontos no grupo intervenção e pelo adequado cegamento dos participantes. Além disso, o período de intervenção foi mais longo do que o da maioria dos ECRs anteriores (15 dias a 8 semanas),^9,10,15,16^ possivelmente proporcionando uma dose terapêutica mais adequada e contribuindo para os efeitos observados.

Para preservar o cegamento dos participantes, as agulhas não foram manipuladas após a inserção em nenhum dos grupos, e os participantes não foram questionados sobre a sensação *De qi*. É pouco provável que essa abordagem tenha influenciado os achados do estudo.

Acredita-se que os efeitos anti-hipertensivos da acupuntura resultem principalmente da modulação do SNC, que desempenha papel importante na regulação do tônus vasomotor simpático e das respostas autonômicas ao estresse.^11,12,43^ Mecanismos adicionais incluem a modulação dos reflexos autonômicos viscerais, a atenuação da atividade simpática eferente, a supressão do SRAA e o aumento da biodisponibilidade de NO, promovendo, assim, vasodilatação e melhora da função endotelial.^6,13,14^

Esses mecanismos podem explicar tanto a redução da PASp quanto a melhora da função endotelial observadas no grupo intervenção, bem como a persistência desses efeitos 30 dias após a conclusão da intervenção.

### Limitações e pontos fortes

A amostra final do estudo foi menor do que o tamanho amostral previsto durante a fase de delineamento, provavelmente porque o recrutamento foi realizado em um centro terciário de referência, onde a maioria dos pacientes apresentava alto risco cardiovascular e já estava recebendo terapia anti-hipertensiva, tornando-os inelegíveis para participação. Apesar do menor tamanho amostral, foram observadas melhorias estatisticamente significativas na PASp e na DMF. A ausência de efeitos significativos sobre a PADp, PASc, PADc e VOP pode ter sido atribuída ao poder estatístico limitado, uma vez que o cálculo do tamanho da amostra foi baseado exclusivamente na detecção de uma diferença mínima entre os grupos de 3 mmHg na PASp. ECRs adicionais com amostras maiores devem avaliar PADp, PASc, PADc e VOP como desfechos primários.

Em comparação com estudos anteriores, nosso ensaio apresenta diversos pontos fortes importantes, incluindo equilíbrio das características basais, procedimentos adequados de randomização e cegamento eficaz dos participantes. Outros pontos fortes incluem a elevada taxa de adesão, o uso de acupuntura simulada não penetrante no grupo controle, a seleção padronizada dos acupontos no grupo intervenção, a duração da intervenção compatível com as recomendações atuais para o manejo não farmacológico da HAS e o emprego de métodos considerados padrão de referência para a avaliação dos desfechos.

Embora este tenha sido um estudo unicêntrico, todas as intervenções foram realizadas pela mesma pesquisadora, medica acupunturiatra com mais de 5 anos de experiência clínica, garantindo consistência dos procedimentos ao longo de todo o ensaio. Além disso, todas as medidas de pressão arterial foram obtidas por meio de dispositivos automatizados validados, minimizando viés de mensuração. A DMF foi avaliada utilizando um sistema de ultrassonografia de alta resolução equipado com braço robótico, e a concordância interavaliadores foi excelente (CCI, 0,94; IC95%, 0,839–0,949; F(29,145), p < 0,001), indicando excelente reprodutibilidade das medidas de DMF entre as visitas do estudo.

## Conclusão

Neste ECR, indivíduos com pré-hipertensão ou HAS estágio 1, de baixo risco cardiovascular e que não estavam recebendo terapia anti-hipertensiva, apresentaram melhora significativa da PASp e da função endotelial após receberem acupuntura duas vezes por semana em oito acupontos predefinidos durante 12 semanas consecutivas. Esses efeitos persistiram por 30 dias após a conclusão do tratamento, sugerindo benefício terapêutico sustentado. Em conjunto, esses achados sugerem que a acupuntura pode representar uma opção promissora de tratamento não farmacológico para essa população. ECRs multicêntricos adicionais, com amostras maiores, são necessários para confirmar esses achados e investigar os efeitos da acupuntura em diferentes estágios da HAS, incluindo pacientes em uso de terapia anti-hipertensiva.

## Material suplementar

Arquivo Suplementar 1

Arquivo Suplementar 2
