# Supplementary material for: Efeitos da Acupuntura na Hipertensão Arterial Sistêmica e na Disfunção Endotelial: Um Ensaio Clínico Randomizado AHEaD
Source: Arq Bras Cardiol. 2026 Aug 3;123(7):e20260462. [Article in Portuguese] doi: 10.36660/abc.20260462 (PMC13668454; doi:10.36660/abc.20260462)
Supplement: Supplementary file 1 [file 0066-782x-abc-123-7-e20260462-suppl01-en.pdf]

Supplement file 1

Table S1. Functions of acupuncture points according to Traditional Chinese Medicine (TCM) theories and their neurophysiological effects.

**Acupoint**	**Function According to TCM**	**Neurophysiological Effects**
PC6 - *Neiguan*	Harmonizes and tonifies the heart (*Xin*); calms the mind.	Sympathetic modulation via action on the hypothalamus, hippocampus, and insula.
LI4 - *Hegu*	Disperses internal heat and excess heart (*Xin*) *Qi*; activates *Qi* and *Xue* circulation in blood vessels.	Attenuates increased sympathetic response.
HT7- *Shenmen*	Harmonizes heart (*Xin*) *Qi*; disperses *Xin* and *Xue* heat; calms the mind.	Autonomic modulation, heart rate reduction, increased heart rate variability.
CV14 - *Juque*	Harmonizes heart (Xin) *Qi*; disperses phlegm-heat from the chest; calms the mind.	Modulation of the limbic system and temporal cortex.
CV17- *Danzhong*	Regulates heat in the upper burner; harmonizes *Qi* circulation; disperses	Increased vagal activity; reduced heart rate.

	damp-heat.	
ST36 - *Zusanli*	Regulates heat between upper and lower burners; tonifies kidney *Qi* (*Shen Qi*), *Yuan Qi*, and *Xue*.	Modulation of cortical excitability in the brain, hippocampus, and limbic system.
LR3 - *Taichong*	Disperses liver (*Gan*) fire; clears heat from the *Xue*; harmonizes and tonifies liver (*Gan*) *Qi* and *Xue*.	Activation of the occipital lobe and regulation of cerebellar areas related to cognition, emotion, and decision-making.
KI3 - *Taixi*	Tonifies kidney *Qi*, *Yin*, and *Xue* (*Shen*).	Activation of the occipital lobe and regulation of cerebellar areas related to cognition, emotion, and decision-making.
