# Supplementary material for: Efeitos da Acupuntura na Hipertensão Arterial Sistêmica e na Disfunção Endotelial: Um Ensaio Clínico Randomizado AHEaD
Source: Arq Bras Cardiol. 2026 Aug 3;123(7):e20260462. [Article in Portuguese] doi: 10.36660/abc.20260462 (PMC13668454; doi:10.36660/abc.20260462)
Supplement: Supplementary file 3 [file 0066-782x-abc-123-7-e20260462-suppl02-en.pdf]

Supplement file 2

Bioethical Aspects

At baseline, all participants were informed of the existence of two groups: one receiving the tested intervention (intervention group) and another not receiving the tested intervention (control group). However, the specific characteristics of the needles used in this study were not disclosed.

Participants with elevated blood pressure at study completion were referred for pharmacological treatment in accordance with the 2020 Brazilian Guidelines on Arterial Hypertension[1].
