# Supplementary material for: Efeitos da Acupuntura na Hipertensão Arterial Sistêmica e na Disfunção Endotelial: Um Ensaio Clínico Randomizado AHEaD
Source: Arq Bras Cardiol. 2026 Aug 3;123(7):e20260462. [Article in Portuguese] doi: 10.36660/abc.20260462 (PMC13668454; doi:10.36660/abc.20260462)
Supplement: Translation

# Translation: Effects of Acupuncture on Arterial Hypertension and Endothelial Dysfunction: The AHEaD Trial

**DOI:** 10.36660/abc.20260462i

**Authors:** Amanda Rodrigues Bitencourt, Ana Luiza Lima Sousa, Paulo Cesar B. Veiga Jardim, Alexandre Massao Yoshizumi, Mikaelle Costa Correia, Priscila Valverde de Oliveira Vitorino, Sandro Rogério Rodrigues Batista, Weimar Kunz Sebba Barroso

**Affiliations:** 1 Brazil Universidade Federal de Goiás – Unidade de Hipertensão Arterial, Goiânia, GO – Brazil; 2 Brazil Hospital Israelita Albert Einstein, São Paulo, SP – Brazil; 3 Brazil Pontifícia Universidade Católica de Goiás – Escola de Ciências Sociais e da Saúde, Goiânia, GO – Brazil

**Keywords:** Hypertension, Vascular Endothelium, Acupuncture

## Abstract

**Background:**

Sympathetic overactivity, oxidative stress, and impaired nitric oxide (NO)-mediated vasodilation are key mechanisms involved in the pathogenesis of hypertension. Acupuncture may decrease blood pressure by modulating autonomic nervous system activity, increasing NO bioavailability, attenuating sympathoexcitatory responses, and promoting vasodilation.

**Objective:**

To evaluate whether acupuncture decreases peripheral systolic blood pressure (pSBP), peripheral diastolic blood pressure (pDBP), central systolic blood pressure (cSBP), central diastolic blood pressure (cDBP), and pulse wave velocity (PWV), and improves flow-mediated dilation (FMD).

**Methods:**

This prospective, single-blind, placebo-controlled, single-center randomized clinical trial included adults with prehypertension or stage 1 hypertension who were at low cardiovascular risk and were not receiving antihypertensive therapy. Participants were randomly assigned to an intervention group receiving manual acupuncture (n = 14) or a control group receiving nonpenetrating sham acupuncture (n = 16). Both groups underwent two 30-minute sessions per week for 12 weeks. The primary outcome was the between-group difference in pSBP measured at baseline (V1), after 12 weeks of treatment (V2), and at the 30-day end-study visit (V3). Statistical significance was defined as p < 0.05.

**Results:**

Baseline characteristics were comparable between the groups. At V2, acupuncture decreased pSBP by 13.2 mmHg (p = 0.003) and increased FMD by 2.7% (p = 0.016). In the control group, pSBP decreased by 4.1 mmHg (p = 0.035), whereas FMD increased by 1.6% (p = 0.029). Compared with the control group, the intervention group demonstrated significantly greater improvements in pSBP (−9.1 mmHg, p = 0.006) and FMD (+3.0%, p = 0.037) at V2. These between-group differences remained significant at V3 (pSBP: −8.4 mmHg, p = 0.038; FMD: +2.9%, p = 0.028).

**Conclusion:**

Acupuncture significantly improved endothelial function and decreased pSBP in adults with prehypertension or stage 1 hypertension who were not receiving antihypertensive therapy after 12 weeks of treatment.

## Introduction

Hypertension is a chronic, multifactorial condition with a high global prevalence and is one of the leading modifiable risk factors for cardiovascular disease (CVD).^1^ Its prevalence increases with age and is highest among individuals older than 60 years.^1^ Additional risk factors include obesity, overweight, physical inactivity, excessive sodium and alcohol consumption, obstructive sleep apnea, and air pollution.^1^

The pathophysiology of hypertension involves complex interactions between genetic and environmental factors that promote sympathetic overactivity and activation of the renin-angiotensin-aldosterone system (RAAS), along with stimulation of central nervous system (CNS) regions, such as the paraventricular nucleus and the rostral ventrolateral medulla (RVLM). These mechanisms increase the release of catecholamines, angiotensin II, aldosterone, and reactive oxygen species (ROS),^2-4^ resulting in enhanced efferent sympathetic activity, increased inflammation, and decreased nitric oxide (NO) bioavailability.^4^ Consequently, vascular contractility is altered, endothelial dysfunction develops, and blood pressure increases.

Increased vascular smooth muscle tone, proliferation of vascular smooth muscle cells within the tunica media, and increased endothelial permeability, which are hallmarks of decreased NO bioavailability, contribute to the vascular remodeling underlying heart, brain, and kidney complications associated with hypertension. Endothelial dysfunction is present in individuals with hypertension even before structural evidence of atherosclerotic disease becomes apparent, making the assessment of endothelial function a valuable clinical tool.^1,4^

Despite the widespread availability of effective antihypertensive medications, hypertension control rates remain suboptimal worldwide, largely because of poor treatment adherence.^1^ Accordingly, nonpharmacological interventions are recommended for all individuals with hypertension.^1,5^

Acupuncture may provide beneficial effects in patients with hypertension.^6-10^ The nociceptive stimulus elicited by needle insertion reaches sympathetic preganglionic neurons in the RVLM, thereby inhibiting visceral sympathoexcitatory reflexes and decreasing vascular smooth muscle spasm.^6,11-14^ Acupuncture also suppresses RAAS-mediated central sympathetic activation,^13^ decreases renin secretion, angiotensin II receptor activity,^12,13^ and ROS production, and activates endothelial NO synthase. These mechanisms increase NO bioavailability, promote vasodilation, and improve endothelial dysfunction.^6,14,15^ Based on these mechanisms,^6,11-14^ we hypothesized that acupuncture could lower blood pressure and improve endothelial function. However, the available evidence remains inconsistent regarding its role as a nonpharmacological treatment for hypertension^10,16^ and its effects on other cardiovascular predictors, including arterial stiffness and endothelial dysfunction.

Accordingly, the current randomized clinical trial (RCT) aimed to evaluate the effects of acupuncture compared with a control group on peripheral systolic blood pressure (pSBP), peripheral diastolic blood pressure (pDBP), central systolic blood pressure (cSBP), central diastolic blood pressure (cDBP), pulse wave velocity (PWV), and flow-mediated dilation (FMD) in patients with prehypertension or stage 1 hypertension after 24 treatment sessions.

## Methods

### Study design and setting

This prospective, single-blind, placebo-controlled, single-center RCT was conducted at the research center of the Unidade de Hipertensão Arterial, Faculdade de Medicina, Universidade Federal de Goiás (UFG), Brazil.

The study was registered in the Brazilian Clinical Trials Registry (under registry code RBR-9qnxb9z) and conducted in accordance with the Revised STandards for Reporting Interventions in Clinical Trials of Acupuncture and CONsolidated Standards Of Reporting Trials guidelines,^17^ and approved by the Human Research Ethics Committee of the Hospital das Clínicas, UFG (approval No. 6.244.770). All participants provided written informed consent before enrollment.

### Study population

The study population comprised adults with prehypertension or stage 1 hypertension who were at low cardiovascular risk and were not receiving antihypertensive therapy.

Exclusion criteria were stage 2 or 3 hypertension; moderate or high cardiovascular risk; use of medications known to affect blood pressure; diabetes mellitus; kidney disease; a history of coronary artery disease and/or cerebrovascular disease; heart failure; thyroid disorders; any condition affecting neural conduction; severe blood dyscrasias; skin lesions or other conditions preventing access to acupoints; pregnancy or lactation; uncontrolled psychiatric disorders; antihypertensive pharmacological treatment within the previous 6 months; or a previous history of acupuncture.

### Sample size calculation

Sample size was calculated for a comparison of mean pSBP between the intervention and control groups, assuming a minimum clinically relevant difference of 3 mmHg. Based on a two-sided significance level of 5%, a statistical power of 90%, and an anticipated dropout rate of 10%, the required sample size was estimated at 25 participants per group.^7^

### Participant recruitment and randomization

Participants were recruited from the research center of the Unidade de Hipertensão Arterial database between March 2024 and February 2025. Individuals who met all inclusion criteria and none of the exclusion criteria were contacted by telephone and invited to attend the baseline visit (V1).

Following enrollment, all participants underwent evaluations at V1, follow-up (V2), and the end-of-study visit (V3), which was conducted 30 days after completion of the intervention.

### Baseline visit

All participants underwent evaluation by the same cardiologist and certified physician acupuncturist, including medical history and physical examination. Hypertension stage was confirmed using the Omron HBP-1100 automated oscillometric device in accordance with the Brazilian Guidelines of Hypertension – 2020.^1^ Individuals with pSBP of 130-159 mmHg and/or pDBP of 85-99 mmHg were considered eligible.

Peripheral blood pressure was measured after 5 minutes of rest, with participants seated, legs uncrossed, and arms supported. Blood pressure was measured in both arms using the Omron HBP-1100 device, and the arm with the higher reading was used for all subsequent measurements, including pSBP, pDBP, cSBP, cDBP, and FMD. Three consecutive measurements were obtained at 1-minute intervals, and the mean of the last two measurements was used to classify hypertension stage. The final values were recorded using a standardized case report form.

After confirmation of eligibility and provision of written informed consent, participants were randomly assigned to either the intervention group (manual acupuncture) or the control group (nonpenetrating sham acupuncture) using a web-based randomization tool.^18^ Allocation was performed consecutively and sequentially at enrollment by an investigator who was not involved in participant recruitment, assessment, or intervention delivery.

Endothelial function, assessed by FMD together with cSBP, cDBP, and PWV, was evaluated in all participants. Assessments were performed by the same cardiologist and certified physician acupuncturist in a quiet, temperature-controlled room between 7:00 and 11:00 a.m. in accordance with current guidelines.^19,20^

Central blood pressure (assessed by cSBP and cDBP) and arterial stiffness (assessed by PWV) were measured using the Mobil-O-Graph^®^ device (IEM GmbH, Stolberg, Germany).^19^

Endothelial function was assessed using the UNEX EF38G high-resolution ultrasound system, the reference standard for measuring FMD. The device incorporates a robotic arm that automatically scans the brachial artery with high precision and reproducibility. A pneumatic cuff positioned distal to the antecubital fossa was inflated to 50 mmHg above the participant’s pSBP for 5 minutes. The longitudinal diameter of the brachial artery was continuously recorded by ultrasound for 60 seconds immediately before cuff inflation and for 2 minutes after cuff deflation.^20^

### Follow-up and end-of-study visits

V2 was conducted after participants completed the 24 intervention sessions. V3 was performed 30 days after V2. At both visits, clinical evaluation and measurements of pSBP, pDBP, cSBP, cDBP, PWV, and endothelial function by FMD were performed according to the same protocol used at V1 by the same investigator.

During V3, participants were asked to identify the type of needle they believed had been used during the intervention, choosing between a skin-penetrating needle (manual acupuncture) and a nonpenetrating needle (Streitberger placebo needle).^21,22^

### Intervention

#### Intervention and control groups

Participants allocated to the intervention group received manual acupuncture using eight sterile stainless-steel needles (0.25 × 30 mm) applied according to the meridian theory of traditional Chinese medicine (TCM),^23^ the recommendations of the Brazilian Medical College of Acupuncture, and the World Health Organization guidelines.^24^ All procedures were performed by a physician acupuncturist with 5 years of clinical experience. Eight acupoints were used: PC6, LI4, HT7, CV14, CV17, ST36, LR3, and KI3 (Figure 1).^25^

**Figure 1:** – Acupoints used in the intervention. A) PC6 (Neiguan) and HT7 (Shenmen); B) LI4 (Hegu); C) CV14 (Juque) and CV17 (Danzhong); D) ST36 (Zusanli); E) LR3 (Taichong); and F) KI3 (Taixi). Adapted from Atlas Gráfico de Acupuntura (ISBN: 9783833156878).

Needles were inserted along the direction of the corresponding meridian at an angle of 30° to 60°, without manual stimulation, and remained in place for 30 minutes per session. Participants were not questioned regarding the needle sensation (De qi). Needle insertion depth generally ranged from 1.0 to 1.5 cm, depending on skin thickness and subcutaneous tissue characteristics. Eight needles were used during each treatment session. Acupoints located on the upper limbs (PC6, LI4, and HT7) and lower limbs (ST36, LR3, and KI3) were applied unilaterally to either the left or right side, with the side randomly selected for each session.

Acupoints were chosen based on their therapeutic relevance for hypertension according to TCM theory,^23^ their neurophysiological effects,^26-30^ and their frequent use in previous RCTs^7,9,15^ (Table S1 in Supplementary File 1).

Participants assigned to the control group received eight nonpenetrating sham acupuncture needles (Streitberger placebo needles)^21,22^ applied by the same physician acupuncturist to eight nonacupuncture points on the trunk and limbs. After placement, the needles remained in position for 30 minutes without manual stimulation. These placebo needles contact the epidermis but do not penetrate the skin or stimulate deeper somatic tissues, as the needle shaft retracts into the handle during application.

All participants underwent 30-minute treatment sessions twice weekly for 12 consecutive weeks (24 sessions in total), with no more than 3 days between sessions. During each session, participants wore an individually assigned eye mask to prevent visual identification of treatment allocation and signed an attendance log. Attendance at more than 80% of the scheduled sessions was considered satisfactory for inclusion in the data analysis.

## Statistical analysis

Statistical analyses were performed using IBM SPSS Statistics for Windows, version 26 (IBM Corp., Armonk, N.Y., USA). Categorical variables are presented as frequencies and percentages, and associations were assessed using the chi-square test or Fisher’s exact test when expected cell counts were ≤ 6. Continuous variables are presented as means and standard deviations. The Shapiro-Wilk test was used to assess the normality of data distribution, and independent-samples Student’s *t* tests were used to compare baseline values between the intervention and control groups.

All analyses were conducted according to the intention-to-treat (ITT) principle, incorporating data from the baseline and follow-up assessments.

A one-way, repeated-measures analysis of variance (ANOVA) was performed to evaluate the effect of the intervention on pSBP, the primary outcome, across the three assessment time points. The assumption of sphericity was evaluated using Mauchly’s test. When the assumption of sphericity was violated (p < 0.05), the Greenhouse-Geisser correction was applied. When a significant main effect was identified, Bonferroni-adjusted post hoc comparisons were performed to determine the time points at which significant differences occurred.

Interrater reliability for FMD measurements was assessed using the intraclass correlation coefficient (ICC) based on a two-way mixed-effects model with absolute agreement.^31^

All statistical tests were two-sided, and statistical significance was defined as p < 0.05. Estimates are presented with 95%CIs. One author had full access to all study data and takes responsibility for the integrity of data and the accuracy of data analysis.

## Ethical considerations

Details are provided in Supplementary File 2.

## Results

Of the 133 individuals screened, 30 met all eligibility criteria and were enrolled in the study, with 16 participants allocated to the control group and 14 to the intervention group. The mean age was 42.0 ± 11.4 years. Baseline characteristics, including sex, body mass index (BMI), physical activity (PA) levels, peripheral and central blood pressure, PWV, and FMD, were comparable between the groups (Table 1).

**Table 1:** – Baseline characteristics of the study participants at V1

Characteristic	Control (n = 16)	Intervention (n = 14)	p-value
**Sex**			0.464*
Female	9 (56.3)	6 (42.9)	
Male	7 (43.8)	8 (57.1)	
**PA**			1.000*
Yes	8 (50.0)	7 (50.0)	
No	8 (50.0)	7 (50.0)	
**BMI**	30.1 ± 4.7	30.1 ± 5.2	0.984^†^
**pSBP, mmHg**	137.3 ± 6.5	137.3 ± 8.6	0.992^†^
**pDBP, mmHg**	86.8 ± 4.4	86.4 ± 6.0	0.812^†^
**cSBP, mmHg**	129.0 ± 12.2	130.5 ± 14.0	0.756^†^
**cDBP, mmHg**	90.1 ± 5.9	92.3 ± 8.5	0.408^†^
**PWV, m/s**	6.7 ± 1.2	6.7 ± 1.1	0.940^†^
**FMD, %**	11.2 ± 3.4	9.9 ± 4.6	0.417^†^

V1: baseline visit; PA: physical activity; BMI: body mass index; pSBP: peripheral systolic blood pressure; pDBP: peripheral diastolic blood pressure; cSBP: central systolic blood pressure; cDBP: central diastolic blood pressure; PWV: pulse wave velocity; FMD: flow-mediated dilation. *p-value from the chi-square test; ^†^p-value from the independent-samples Student’s t test. Data are presented as mean ± SD or n (%) as appropriate.

All participants completed the evaluations at V1, V2, and V3. Session attendance rates were 99.7% in the intervention group and 94.7% in the control group. Only one participant had an attendance rate (54%) below the prespecified threshold (> 80%) but was retained in the analysis according to the ITT principle.

All participants in the intervention group believed they had received skin-penetrating needles. In the control group, only two participants correctly identified that they had received nonpenetrating needles; excluding these participants did not alter the study findings. No serious adverse events were reported. One participant in the intervention group reported mild pain at the needle insertion sites.

BMI and PA levels remained comparable between the groups at V1, V2, and V3.

Figure 2 and Figure 3 present the main findings. Both groups showed decreases in pSBP after the 12-week intervention; however, the decrease was significantly greater in the intervention group than in the control group. Moreover, the intervention group maintained this decrease at V3, whereas no sustained effect was observed in the control group. Likewise, FMD increased significantly in the intervention group compared with the control group after the intervention, and this improvement was maintained at V3 (Central Illustration ).

**Figure 2:** – Mean pSBP by study group at V1, V2, and V3. Between-group differences in mean pSBP are shown for V2 (A) and V3 (B). *p-values were obtained using repeated-measures ANOVA with Bonferroni correction. ANOVA: analysis of variance; CG: control group; IG: intervention group; pSBP: peripheral systolic blood pressure; V1: baseline visit; V2: follow-up visit; V3: 30-day end-study visit.

**Figure 3:** – Comparison of mean FMD by study group at V1, V2, and V3. Between-group differences in mean FMD are shown for V2 (A) and V3 (B). *p-values were obtained using repeated-measures ANOVA with Bonferroni correction. ANOVA: analysis of variance; CG: control group; FMD: flow-mediated dilation; IG: intervention group; V1: baseline visit; V2: follow-up visit; V3: 30-day end-study visit.

At V2, participants in the intervention group exhibited a 13.2 mmHg decrease in pSBP and a 2.7% increase in FMD. In contrast, the control group showed a 4.1 mmHg decrease in pSBP and a 1.6% increase in FMD. Between-group comparisons at V2 demonstrated significantly greater improvements in the intervention group for pSBP (−9.1 mmHg) and FMD (+3.0%). At V3, the intervention group maintained significantly greater improvements in pSBP (−8.4 mmHg) and FMD (+2.9%) than the control group (Table 2).

**Table 2:** – Comparison of mean values between and within study groups at V1, V2, and V3

Characteristic	Control (n = 16)	p-value* (within group)	Intervention (n = 14)	p-value* (within group)	p-value^**†**^ (between groups)
**pSBP**		0.018			
V1	137.3 ± 6.5		137.3 ± 8.6		0.992
V2	133.2 ± 10.1	0.035^a^	124.1 ± 5.8	0.003^a^	0.006
V3	135.3 ± 11.3	1.000^b^	126.9 ± 9.4	0.017^b^	0.038
**pDBP**		0.751			
V1	86.8 ± 4.4		86.4 ± 6.0		0.812
V2	82.2 ± 7.4	0.003^a^	81.6 ± 6.5	0.002^a^	0.823
V3	84.7 ± 9.4	0.760^b^	83.0 ± 5.9	0.147^b^	0.559
**cSBP**		0.439			
V1	129.0 ± 12.2		130.5 ± 14.0		0.756
V2	126.2 ± 13.1	0.973^a^	123.0 ± 9.2	0.277^a^	0.453
V3	125.9 ± 10.5	0.793^b^	128.6 ± 14.8	1.000^b^	0.576
**cDBP**		0.728			
V1	90.1 ± 5.9		92.3 ± 8.5		0.408
V2	86.2 ± 8.1	0.197^a^	88.7 ± 7.3	0.418^a^	0.390
V3	86.0 ± 8.0	0.097^b^	90.1 ± 9.3	1.000^b^	0.199
**PWV**		0.083			
V1	6.7 ± 1.2		6.7 ± 1.1		0.940
V2	6.7 ± 1.3	1.000^a^	6.4 ± 1.0	0.056^a^	0.502
V3	6.7 ± 1.3	1.000^b^	6.6 ± 1.3	1.000^b^	0.873
**FMD**		< 0.001			
V1	11.2 ± 3.4		9.9 ± 4.6		0.417
V2	9.6 ± 2.3	0.029^a^	12.6 ± 4.5	0.016^a^	0.037
V3	9.1 ± 2.6	0.114^b^	12.0 ± 4.0	0.098^b^	0.028

V1: baseline visit; V2: follow-up visit; V3: 30-day end-study visit; pSBP: peripheral systolic blood pressure; pDBP: peripheral diastolic blood pressure; cSBP: central systolic blood pressure; cDBP: central diastolic blood pressure; PWV: pulse wave velocity; FMD: flow-mediated dilation; ANOVA: analysis of variance. *p-values from repeated-measures ANOVA with Bonferroni correction for comparisons between V1 and V2 (a) and between V1 and V3 (b); †p-values from repeated-measures ANOVA with Bonferroni correction for between-group comparisons. Intervention and control data are presented as mean ± SD.

A significant group × time interaction was observed for pSBP, which indicates that changes over time differed between the intervention and control groups. A significant group × time interaction was also observed for FMD (Table 2).

Repeated-measures ANOVA demonstrated a significantly greater decrease in pSBP across the three assessment time points in the intervention group than in the control group (intervention group: F = 10.964, p = 0.006, η^2^ = 0.458; control group: F = 2.276, p = 0.120, η^2^ = 0.132).

## Discussion

The findings of this RCT demonstrated significant improvements in pSBP and FMD after 12 weeks of intervention, with these effects persisting 30 days after treatment cessation. Although the primary objective was to evaluate the effects of acupuncture on blood pressure rather than clinical outcomes, the observed improvements in pSBP and FMD may have prognostic relevance, consistent with previous studies.^4,20,32^ A 5-mmHg decrease in pSBP has been associated with approximately 10% and 8% decreases in the risk of major cardiovascular events and ischemic heart disease, respectively.^32^

Flachskampf et al.^9^ reported significant decreases in 24-hour ambulatory systolic and diastolic blood pressure (−6.4 and −3.7 mmHg, respectively; p < 0.001), as well as in daytime pSBP (−7.3 mmHg; p < 0.001), among 72 patients with stage 1 or 2 hypertension who underwent 22 acupuncture sessions over 6 weeks at three bilateral acupoints, compared with 68 patients who received penetrating sham acupuncture. Although 75% of participants were receiving stable antihypertensive therapy, a subgroup analysis of 35 participants who were not receiving pharmacological treatment (19 in the acupuncture group) demonstrated sustained decreases in the 24-hour mean blood pressure (−6.6 mmHg for pSBP, p = 0.018; and −3.7 mmHg for pDBP, p = 0.027). Similarly, acupuncture decreased pSBP in the current study. However, unlike Flachskampf et al.,^9^ we did not perform 24-hour ambulatory blood pressure monitoring, did not observe a significant effect on pDBP, enrolled only participants who were not receiving antihypertensive therapy, and evaluated a longer intervention period (12 weeks). In addition, we used a validated nonpenetrating sham acupuncture procedure designed to minimize physiological effects, and all participants in the intervention group received treatment at the same eight predefined acupoints.

Similarly, Liu et al.^7^ reported a decrease in mean pSBP (−8.6 mmHg; p = 0.031) 4 weeks after completion of an 8-week acupuncture intervention in 15 patients with prehypertension or stage 1 hypertension compared with untreated controls. Consistent with these findings, we observed a sustained decrease in mean pSBP in the intervention group 30 days after completion of the intervention (−8.4 mmHg; p = 0.038). Despite differences in study design and outcome assessment, the sustained decrease in blood pressure observed after treatment cessation in both studies supports the hypothesis that acupuncture may produce persistent antihypertensive effects.

The mean between-group decrease in pSBP of 9.1 mmHg observed with acupuncture may be clinically meaningful as it is comparable to the decreases reported in RCTs evaluating antihypertensive monotherapy.^33,34^ A systematic review of 46 RCTs comparing nine angiotensin receptor blockers with placebo reported decreases in pSBP of 6-10 mmHg.^33^ Likewise, a meta-analysis of 60 RCTs found a mean decrease of 9.1 mmHg in pSBP with thiazide diuretics compared with placebo.^34^

The decrease in pSBP observed in the control group (−4.1 ± 10.1 mmHg; p = 0.035) may be attributable at least in part to a placebo effect which is expected in virtually any therapeutic intervention. The magnitude of this effect depends on several factors, including the mode of administration, treatment setting, and the therapeutic relationship between the participant and the practitioner.^35^ Because both groups underwent treatment under identical conditions (eg, the same clinical setting, practitioner, treatment schedule, number of sessions, and number of stimulation sites) with the only difference being the use of penetrating versus nonpenetrating needles, a comparable placebo effect would be expected in both groups. Accordingly, the significantly greater decrease in mean pSBP in the intervention group than in the control group (−9.1 mmHg; p = 0.006), together with the greater improvement in endothelial function, suggests that the therapeutic effects of acupuncture extended beyond those attributable to placebo.

Although FMD assessment is not routinely performed in clinical practice, it is a valuable tool for evaluating the short- and long-term effects of both pharmacological and nonpharmacological interventions. Improved FMD reflects enhanced endothelial function and is associated with protection against atherosclerotic disease and CVD.^36^

Consistent with the findings of Park et al.,^15^ our results demonstrated improved endothelial function following acupuncture, potentially mediated by enhanced endothelial NO synthase activation and NO production,^14^ together with modulation of sympathetic activity.^11,12^ In their study evaluating the acute effects of four acupuncture sessions at ST36 alone or combined with PC6 on FMD in 15 patients with hypertension receiving antihypertensive therapy, Park et al.^15^ reported a significantly greater increase in FMD in the acupuncture group than in the placebo group. However, unlike our study, penetrating needles were used in the control group, and FMD was assessed over a shorter period after brachial artery reactive hyperemia (60 seconds).

To the best of our knowledge, the AHEaD trial is the first study to demonstrate sustained improvements in endothelial function following acupuncture using the same predefined acupoints for all participants, a nonpenetrating sham acupuncture control, and FMD measurements obtained with a high-resolution ultrasound system equipped with an automated robotic arm, the reference standard for assessing FMD.^20^

Brachial artery FMD is an independent predictor of CVD,^4,20^ and persistently impaired FMD despite optimized treatment is associated with an increased risk of future cardiovascular events.^36^ Therefore, the improvement in FMD observed after acupuncture may have important clinical implications. A meta-analysis showed that each 1% increase in brachial artery FMD is associated with an 8%-13% decrease in CVD risk.^36^

The decrease in pSBP observed after acupuncture in the current study contrasts with the findings of the SHARP trial.^16^ In SHARP, 12 acupuncture sessions administered over 6-8 weeks in patients with stage 1 or 2 hypertension did not significantly decrease pSBP compared with sham acupuncture. Approximately 70% of participants in that trial had previously received antihypertensive therapy, which was discontinued before randomization, and the sham intervention involved skin-penetrating needles. In contrast, our study used nonpenetrating sham needles, thereby eliminating nociceptive stimulation associated with deep tissue penetration. In addition, our intervention included a greater number of treatment sessions, enrolled participants who had not received antihypertensive therapy within the previous 6 months, and extended over 12 weeks, consistent with current guideline recommendations for nonpharmacological management of hypertension.^1,5^

Terenteva et al.^37^ reported improvements in central blood pressure and arterial stiffness parameters after 8 weeks of acupuncture (three sessions per week) using seven bilateral acupoints in 23 participants, despite the absence of participant blinding and a placebo control group. In contrast, acupuncture did not improve arterial stiffness parameters in the current study. This discrepancy may be explained by differences in treatment frequency (two versus three sessions per week), the number of acupoints used, and the relatively small sample size of our study. In hypertension, arterial stiffening results from complex molecular, cellular, and mechanical processes that impair arterial distensibility and increase intima-media thickness.^19^ Acupuncture may exert a greater effect on endothelial function than on the vascular smooth muscle remodeling associated with fibrosis and arterial stiffening by increasing NO bioavailability and promoting vasodilation. Additional studies are warranted to clarify the effects of acupuncture on arterial stiffness.

Nonpharmacological treatment is recommended for all individuals with hypertension.^1,5^ Sodium and alcohol restriction, adherence to a healthy dietary pattern, regular PA, and spirituality-based interventions have been shown to decrease pSBP by 5.5-12.0 mmHg.^38-41^ In the current study, acupuncture produced decreases in pSBP and improvements in FMD of a magnitude comparable to those reported for other nonpharmacological interventions, suggesting that acupuncture may represent an additional nonpharmacological treatment option for hypertension.

In RCTs, poor adherence to the intervention may compromise the validity and interpretation of the findings.^42^ In the current study, adherence was high in both groups, with no significant between-group differences. This likely reflects the fact that all treatment sessions were delivered by the same investigator, an experienced clinician specializing in hypertension management, who also offered flexible scheduling. The high adherence rate, together with the maintenance of comparable baseline characteristics throughout the study for variables that could influence blood pressure, supports the interpretation that the observed effects were attributable to the intervention.

The between-group decrease in mean pSBP observed in the intervention group (−9.1 mmHg; p = 0.006) exceeded that reported in a previous meta-analysis.^10^ This finding may be explained by the high adherence rate, the use of nonpenetrating sham needles in the control group, a standardized acupoint protocol in the intervention group, and appropriate participant blinding. Furthermore, the intervention period was longer than that of most previous RCTs (15 days to 8 weeks),^9,10,15,16^ potentially providing a more adequate treatment dose and contributing to the observed effects.

To preserve participant blinding, the needles were not manipulated after insertion in either group, and participants were not asked about the De qi sensation. This approach was unlikely to have influenced the study findings.

The antihypertensive effects of acupuncture are thought to result primarily from modulation of the CNS, with an important role in regulating sympathetic vasomotor tone and autonomic responses to stress.^11,12,43^ Additional mechanisms include modulation of visceral autonomic reflexes, attenuation of sympathetic outflow, suppression of the RAAS, and increased NO bioavailability, thereby promoting vasodilation and improving endothelial function.^6,13,14^

These mechanisms may explain both the decrease in pSBP and the improvement in endothelial function observed in the intervention group as well as the persistence of these effects 30 days after completion of the intervention.

### Limitations and strengths

The final study sample was smaller than the target sample size estimated during the design phase, likely because recruitment was conducted at a tertiary referral center, where most patients had high cardiovascular risk and were already receiving antihypertensive therapy, rendering them ineligible for participation. Despite the smaller sample size, statistically significant improvements were observed in pSBP and FMD. The absence of significant effects on pDBP, cSBP, cDBP, and PWV may have been attributable to limited statistical power, as the sample size calculation was based exclusively on detecting a minimum between-group difference of 3 mmHg in pSBP. Further RCTs with larger sample sizes should evaluate pDBP, cSBP, cDBP, and PWV as primary outcomes.

Compared with previous studies, our trial has several important strengths, including balanced baseline characteristics, appropriate randomization procedures, and effective participant blinding. Additional strengths include the high adherence rate, the use of nonpenetrating sham acupuncture in the control group, standardized acupoint selection in the intervention group, an intervention duration consistent with current recommendations for nonpharmacological management of hypertension, and the use of reference-standard methods for outcome assessment.

Although this was a single-center study, all interventions were performed by the same investigator, a physician acupuncturist with more than 5 years of clinical experience, thereby ensuring procedural consistency throughout the trial. In addition, all blood pressure measurements were obtained using validated automated devices, minimizing measurement bias. FMD was assessed using a high-resolution ultrasound system equipped with a robotic arm, and interrater agreement was excellent (ICC, 0.94; 95%CI, 0.839-0.949; F(29,145), p < 0.001), which indicates excellent reproducibility of FMD measurements across study visits.

## Conclusion

In this RCT, individuals with prehypertension or stage 1 hypertension who were at low cardiovascular risk and were not receiving antihypertensive therapy experienced significant improvements in pSBP and endothelial function after receiving acupuncture twice weekly at eight predefined acupoints for 12 consecutive weeks. These effects persisted for 30 days after treatment completion, which suggests a sustained therapeutic benefit. Collectively, these findings suggest that acupuncture may represent a promising nonpharmacological treatment option for this population. Further multicenter RCTs with larger sample sizes are warranted to confirm these findings and to investigate the effects of acupuncture across different stages of hypertension, including among patients receiving antihypertensive therapy.

## Supplemental Materials

Supplement file 1

Supplement file 2
